# The Role of Permissive and Induced Hypotension in Current Neuroanesthesia Practice

**DOI:** 10.3389/fsurg.2017.00001

**Published:** 2017-01-30

**Authors:** Suren Soghomonyan, Nicoleta Stoicea, Gurneet S. Sandhu, Jeffrey J. Pasternak, Sergio D. Bergese

**Affiliations:** ^1^Department of Anesthesiology, The Ohio State University Wexner Medical Center, Columbus, OH, USA; ^2^Department of Anesthesiology, Mayo Clinic College of Medicine, Rochester, MN, USA; ^3^Department of Neurological Surgery, The Ohio State University Wexner Medical Center, Columbus, OH, USA

**Keywords:** permissive hypotension, anesthesia for neurosurgery, intraoperative blood pressure, controlled hypotension, intraoperative cerebral ischemia, intraoperative blood loss

## Abstract

**Background:**

Induced hypotension (IH) had been used for decades in neurosurgery to reduce the risk for intraoperative blood loss and decrease blood replacement. More recently, this method fell out of favor because of concerns for cerebral and other end-organ ischemia and worse treatment outcomes. Other contributing factors to the decline in its popularity include improvements in microsurgical technique, widespread use of endovascular procedures, and advances in blood conservation and transfusion protocols. Permissive hypotension (PH) is still being used occasionally in neurosurgery; however, its role in current anesthesia practice remains unclear. Our objective was to describe contemporary utilization of IH and PH (collectively called PH) in clinical practice among members of the Society for Neuroscience in Anesthesiology and Critical Care (SNACC).

**Methods:**

A questionnaire was developed and distributed among SNACC members that addressed practice patterns related to the use of PH. The responses were analyzed based on the number of individuals who responded to each specific question.

**Results:**

Of 72 respondents, 67.6% reported over 10 years of clinical experience, while 15.5% reported 5–10 years of post-training experience. The respondents admitted to providing anesthesia for 300 (median) neurosurgical cases per year. PH was applied most commonly during open interventions on cerebral aneurysms (50.8%) and arteriovenous malformations (46%). Seventy-three percent of respondents were not aware of any complications in their practice attributable to PH.

**Conclusion:**

PH is still being used in neuroanesthesia practice by some providers. Further research is justified to clarify the risks and benefits of PH in modern neuroanesthesia practice.

## Introduction

In 1918, Canon and his colleagues introduced the concept of permissive hypotension (PH) as a resuscitation strategy used in the acute phase of traumatic hemorrhagic shock (as cited in ref. 1).

Permissive hypotension and its variation known as controlled or induced hypotension (IH) were used in neurosurgical practice for decades to reduce intraoperative blood loss, create a clearer surgical view, and relax the aneurysmal neck to facilitate clipping. The main distinction between PH and IH is mostly quantitative. PH (formerly known as moderate level of controlled hypotension) usually refers to an intraoperative blood pressure (BP) reduction of no more than 20–30% of baseline values. Usually, it can be achieved by adjustments in anesthesia without using vasoactive drugs. IH, on the other hand, commonly refers to a deeper level of controlled BP decrease requiring titrated infusion of vasoactive medications. Potential hazards of these methods include increased risk of cerebral ischemia, retraction injury, inadequate local hemostasis with risk of rebleeding, cardiovascular complications, postoperative renal dysfunction, and others. Additional contributing factors to decreased popularity of both methods were improvements in microsurgical technique and equipment, routine use of temporary clipping of the feeding vessels during dissection, and mobilization of the aneurysmal neck as well as significant advances in endovascular techniques, anesthesia, and neuroimaging methods. As a result, IH subsequently fell out of favor. PH, on the other hand, was not abandoned completely and is still being applied with uncertain frequency worldwide mostly being advocated by surgeons.

It would be useful and of practical interest to determine the practice patterns of PH (and IH) in modern day neurosurgical anesthesia.

The main objective of the study was to conduct a survey to determine the role of PH (and IH) in clinical practice among Society for Neuroscience in Anesthesiology and Critical Care (SNACC) members and analyze practical aspects of various approaches to the method. In an attempt to reduce the variability of responses, which would make data analysis more problematic, we used the term PH for both paths of intraoperative controlled BP reduction.

## Participants and Methods

The survey was approved by the Institutional Review Board of the Ohio State University Wexner Medical Center.

A 26-item questionnaire that addressed the principle aspects of PH was created and sent to SNACC to be forwarded to its active members. SNACC announced *via* emails the upcoming survey encouraging its members to participate. The survey was conducted for 4 weeks during October–November of 2014. SNACC distributed the questionnaire among its members and used Survey Monkey to collect anonymous responses and conduct a preliminary analysis of the results. These results were then forwarded to our team to complete the data analysis. Not all respondents answered all questions, so all calculations were made based on the actual number of respondents for each question. No detailed statistical tests were applied considering survey specifics and variability of answers.

## Results

Overall, 72 participants (13% of SNACC members) completed the survey. The results are presented in Table 1 and Figures 1–11.

**Table 1:** **Twenty-six-item questionnaire and results of the conducted survey**.

Question	Answer	Responses (%)	*N* responded	Respondents (total)
1. What is the country of your home institution?	Figure 1			68

2. Do you use “permissive” or “induced” hypotension during any of the following surgeries?				63
3. If you responded in the previous question that you use permissive or induced hypotension (IH) during “other procedures,” please state the types of procedures	Figure 5			5

4. Do you use invasive arterial BP monitoring (arterial line) when you perform permissive or IH?	Always Sometimes Never	87.3 9.5 3.2	55 6 2	63

5. Where do you locate the pressure transducer in patients who are not in a horizontal position if an arterial line is used?	At the heart level At the ear level	16.9 83.1	11 54	65

6. Do you use SBP (versus mean blood pressure) as a guide in cases where you perform permissive or IH?	Always Sometimes Never	17.2 50.0 32.8	11 32 21	64

7. If you use systolic arterial pressure as a guide, what is your target during permissive or IH in non-hypertensive patients?	Figure 6			47

8. Do you use mean arterial BP as a guide during permissive or IH?	Always Sometimes Never	47.7 43.1 9.2	31 28 6	65

9. If you use mean arterial pressure as a guide, what is your target during permissive or IH in non-hypertensive patients?	Figure 7			60

10. What drugs do you use to reach the target BP value?	Figure 11			66

11. Do you use any devices during surgery with permissive or IH to monitor the integrity of cerebral perfusion?	Figure 10			50

12. Do you perform permissive or IH in patients with chronic hypertension?	Always Sometimes Never	2.9 76.5 20.6	2 52 14	68

13. If you perform permissive or IH in hypertensive patients, do you change the minimum target BP with respect to non-hypertensive patients?	Yes No I do not use permissive hypotension in hypertensive patients	80.0 4.6 15.4	52 3 10	65

14. If you answered “YES” to the last question, please mark the option that best fits your practice	Tolerate decreases in systolic or mean pressure to 20% of basal values Tolerate decreases in systolic or mean pressure to 30% of basal values Tolerate decreases in systolic or mean pressure to 40% of basal values	50.0 46.3 3.7	27 25 2	54

15. Are you aware of any complications in your patients treated with intraoperative permissive or IH that may be associated with this technique?	Figure 8			67

16. If you answered “YES” to the prior question, please specify the complication	Figure 9			19

17. In your opinion, which of the following conditions are contraindications for permissive or IH?	Uncontrolled arterial hypertension Renal insufficiency Coronary artery disease Carotid insufficiency	70.3 50.0 73.4 89.1	45 32 47 57	64

18. Do you use permissive or IH during procedures when the patient is NOT in the horizontal position?	Yes No	32.4 67.7	22 46	68

19. If the answer was “YES” to the prior question, please specify the other positions many respondents reported more than one position	Head up Any position requested by surgeon Beach Chair Prone Park bench Lateral Sitting/semi-sitting Supine with back up Spine surgery Positioning for shoulder surgery (will keep SBP ≥ 100 mmHg)	27.3 4.5 9.1 18.2 27.3 13.6 4.5 4.5 4.5 4.5	6 1 2 4 6 3 1 1 1 1	22

20. Do you use permissive or IH in combination with other methods to prevent intraoperative blood loss (e.g., hemodilution)?	Yes No	14.5 85.5	10 59	69

21. If you answered “YES” to the prior question, what techniques do you use?	Propofol and remifentanil (not in neurosurgery)	14.3	1	7
	Hemodilution	14.3	1	
	Low-dose adrenalin (epinephrine) infusion “to maintain the cardiac output and peripheral tissue perfusion”	14.3	1	
	Tranexamic acid	28.6	2	
	Methods to improve venous return (unspecified)	14.3	1	
	Adenosine	14.3	1	

22. Do you use any special techniques to decrease the risk of side effects of permissive or IH (e.g., limiting the duration)?	I never use permissive hypotension Yes No	11.6 46.4 42.0	8 32 29	69

23. If you answered “YES” to the prior question, what techniques do you use?	Hydration	6.3	2	32
	Maintaining adequate hematocrit	9.4	3	
	Additional monitoring (A-line, NIRS, neuromonitoring, EEG burst suppression, SSEP)	15.6	5	
	Limit duration (a few minutes to less than 2 h)	62.5	20	
	Limit the extent of hypotension and adjust BP targets	18.8	6	
	Operated by experienced surgeon	3.1	1	
	Using only at the most critical points of surgery (clipping of the aneurysm, aneurysmal rupture, difficulty in controlling bleeding, post-resection in AVMs, pedicle screw placement in spinal surgery, when requested by the surgeon)	12.5	4	
	low-dose adrenalin (epinephrine) infusion to maintain the cardiac output and peripheral tissue perfusion	3.1	1	
	Passive patient cooling or induced hypothermia	6.3	2	
	Increasing FiO2	3.1	1	
	Deep anesthesia or pharmacological neuroprotection	9.4	3	
	Let the patient recover hemodynamically before re-dosing adenosine	3.1	1	
	Limit the decrease in MAP to 30% below baseline for 10–25 min limit drastic decreases to 3–5 min	3.1	1	32

24. For how many years have you worked in the field of anesthesia?	Figure 2			71

25. Approximately how many neurosurgical cases require general anesthesia per year in your hospital or institution?	Figure 3			66

26. Approximately how many general anesthetics do YOU provide per year for neurosurgical cases?	Figure 4			68

*SBP, systolic blood pressure; NIRS, near-infrared spectrometry; EEG, electroencephalography; SSEP, somatosensory evoked potentials; BP, blood pressure; AVM, arteriovenous malformation; MAP, mean arterial pressure*.

**Figure 1:**
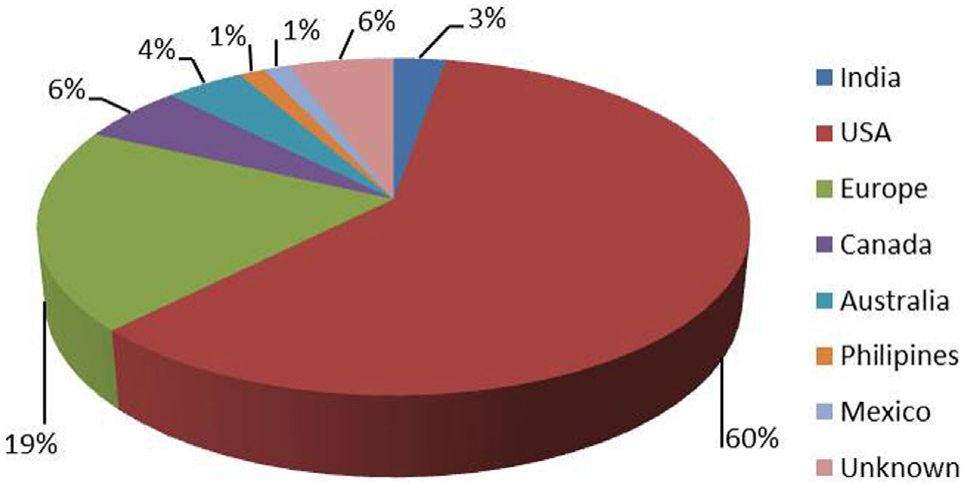
**Geographical representation of respondents (68 respondents)**.

**Figure 2:**
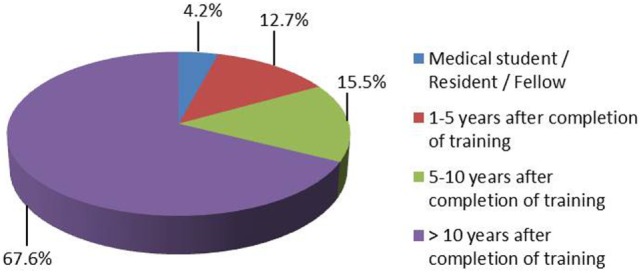
**Experience of the respondents in the field of clinical anesthesia (71 respondents)**.

**Figure 3:**
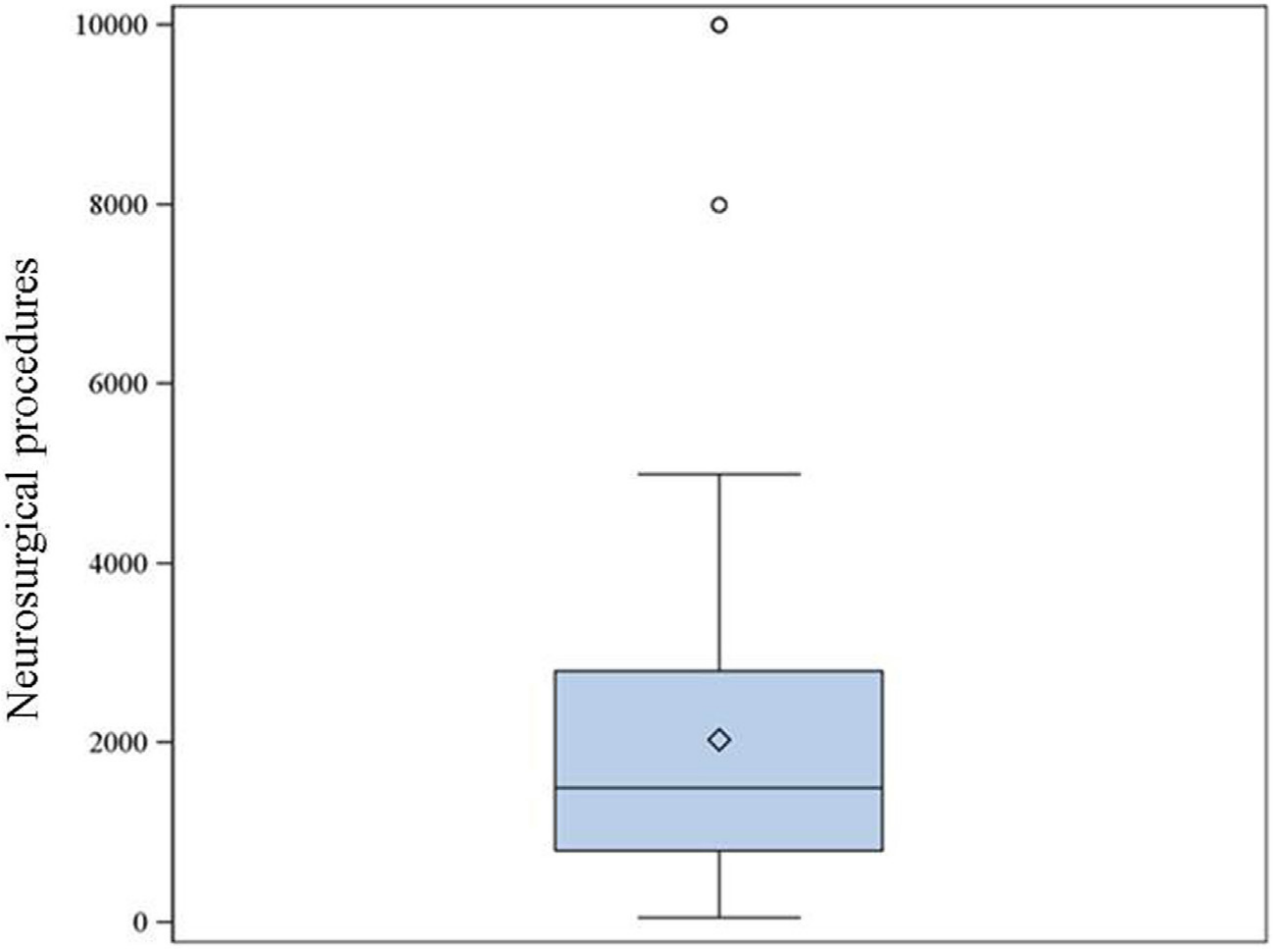
**The reported number of neurosurgical procedures under general anesthesia per year in the institutions where the respondents work (66 respondents)**. Median: 1,500, 25–75% range: 800–2,800.

**Figure 4:**
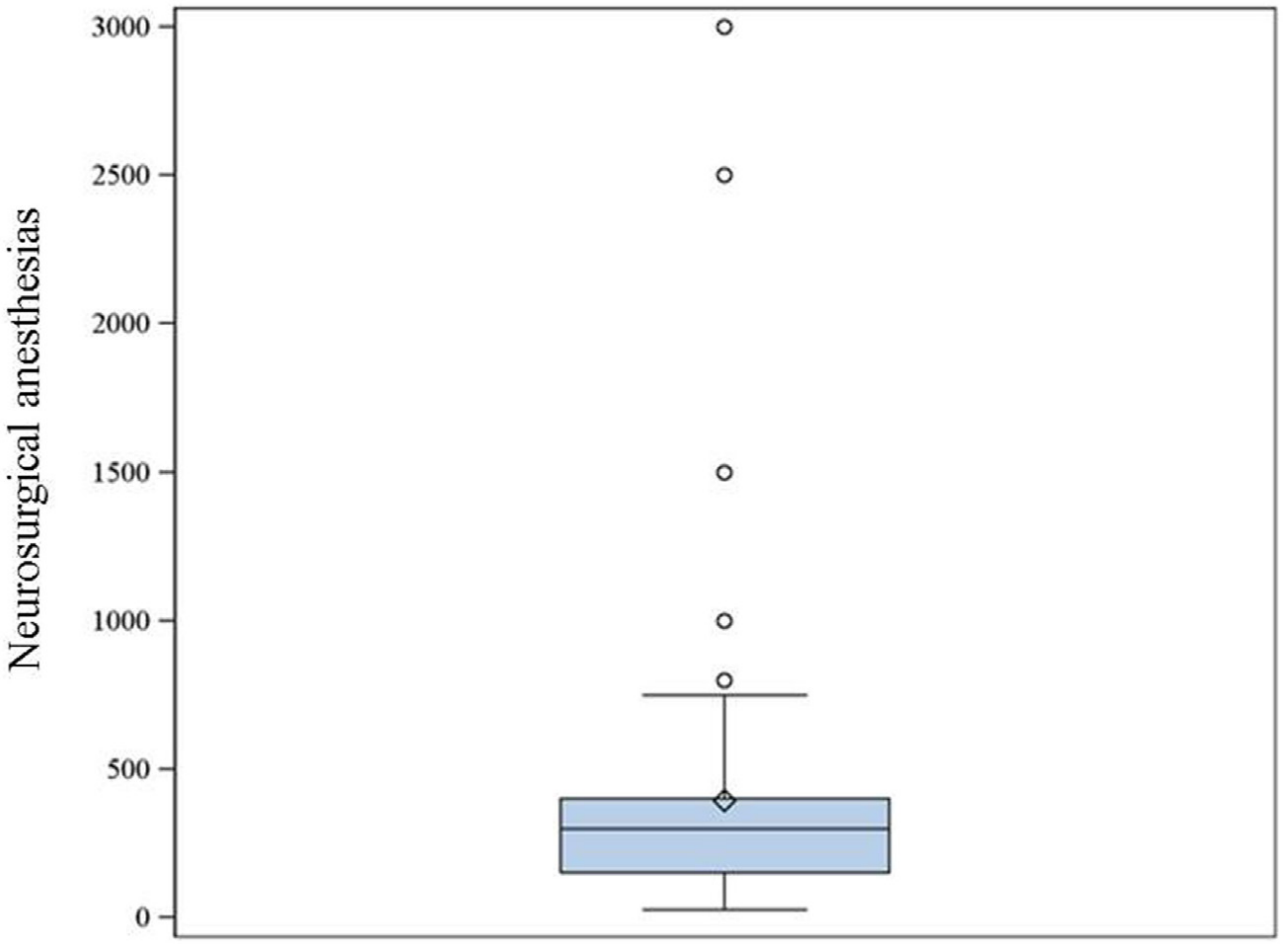
**The reported number of neurosurgical procedures per year during which the respondents provided general anesthesia (68 respondents)**. Median: 300, 25–75% range: 150–400.

**Figure 5:**
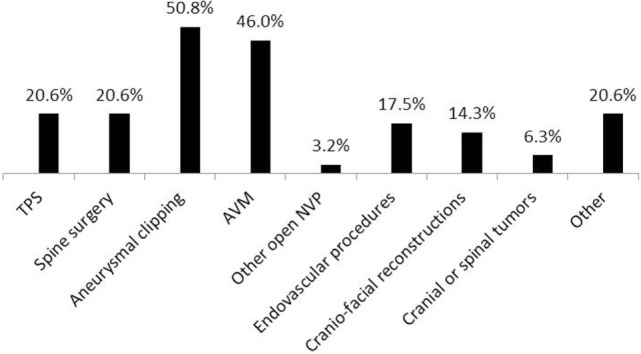
**Procedures when permissive hypotension (PH) had been used (63 respondents)**. TPS, transsphenoidal pituitary surgery; AVM, arteriovenous malformations; NVP, neurovascular procedures; other—skull base and acoustic neuroma surgery, unspecified time periods and stages during open aneurysm surgeries, endovascular embolization, cases when adenosine-induced cardiac arrest was applied, unspecified cases when PH was requested by surgeon and when the method was deemed appropriate.

**Figure 6:**
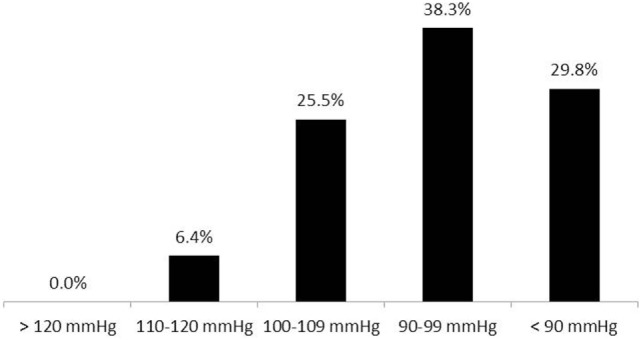
**Systolic blood pressure target values during anesthesia among non-hypertensive patients (47 respondents)**.

**Figure 7:**
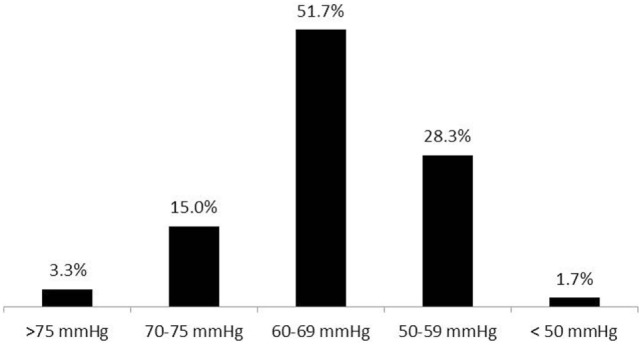
**Mean blood pressure target values during anesthesia among normotensive patients (60 respondents)**.

**Figure 8:**
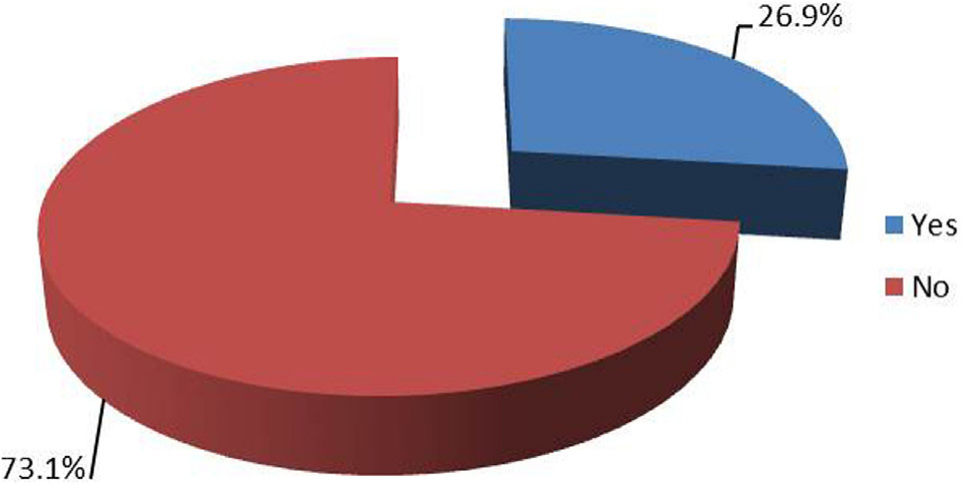
**Seventy-three percent of respondents were not aware of any complications among their patients treated with intraoperative permissive or induced hypotension that could be attributed to the technique**. Twenty-seven percent of respondents were aware of such cases in their practice (67 respondents).

**Figure 9:**
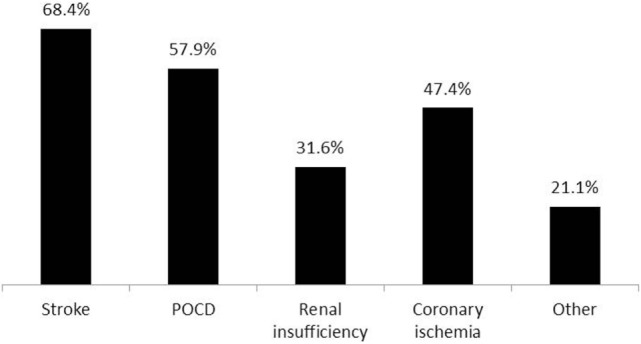
**Awareness of specific adverse effects related to intraoperative permissive hypotension in their practice among respondents (19 respondents)**. Stroke (13), POCD—postoperative cognitive dysfunction (11), renal insufficiency (6), coronary ischemia (9), other—possible cognitive dysfunction (1), rhabdomyolysis (1), postoperative bleeding (1), postoperative visual loss (1).

**Figure 10:**
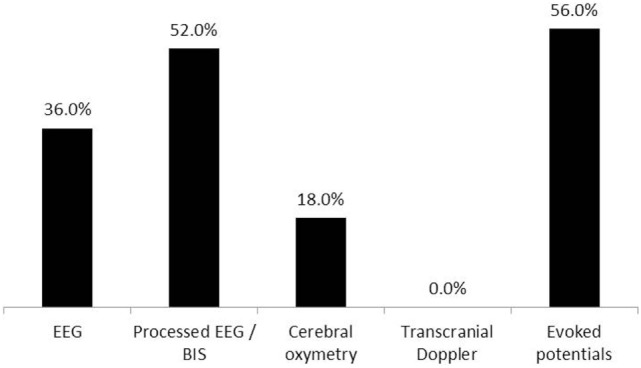
**Modalities of monitoring used during intraoperative permissive hypotension**. Electroencephalography (EEG)—encephalography, processed EEG/BIS—processed encephalography/bispectral index. Fifty respondents (three non-relevant responses were excluded).

**Figure 11:**
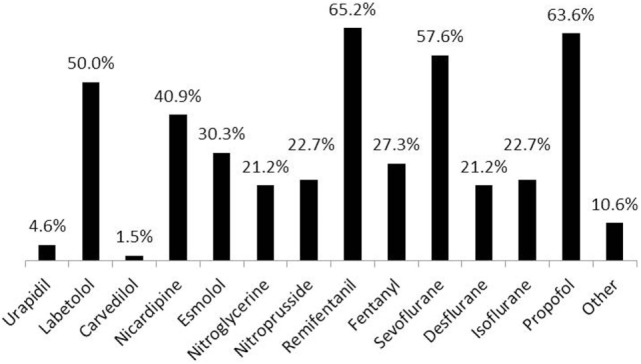
**Drugs used to reach target blood pressure (BP) values during permissive hypotension (66 respondents)**. Other drugs included: magnesium, clevidipine, phentolamine, dexmedetomidine (two respondents), adenosine, and all of the listed medications (one respondent).

Eighty-nine percent of respondents represented the USA, Canada, the European continent, and Australia (Figure 1). The majority of respondents (83.1%) stated to have at least 5 years of clinical experience in neurosurgical anesthesiology (Figure 2). According to responses, the participating anesthesiologists managed 300 (median) neurosurgical cases per year and worked in hospitals covering neurosurgical procedures under anesthesia for approximately 1,500 patients per year (Figures 3 and 4). The intraoperative PH had been reportedly used by 50.8% during open aneurysmal clipping, while 46% applied the method during surgery on arteriovenous malformations (AVM) (Figure 5). The method was applied less frequently with other procedures. Among respondents, 67.2% of anesthesiologists used systolic blood pressure (SBP) value to conduct PH and up to 70.2% of them maintained the SBP level above 90 mmHg. Less frequently (29.8% of respondents), SBP target values <90 mmHg were used (Figure 6). More commonly (90.8%), the mean blood pressure (MBP) was used to control the level of hypotension, and 70% preferred maintaining the MBP ≥60 mmHg (Figure 7).

Seventy-three percent of respondents were not aware of any adverse effects encountered during their practice that could directly be attributed to the use of PH in their patients (Figure 8). The reported adverse effects of PH are shown in Figure 9.

Most commonly, raw recordings or processed electroencephalography (EEG), evoked potentials (EP), and cerebral oximetry were used for intraoperative neurophysiological monitoring during PH (Figure 10).

The majority of anesthesiologists (79.4%) reported using PH in patients with chronic arterial hypertension, and most of them tolerated 20–30% decrease in SBP or MBP during the procedure.

One-third of the respondents applied PH when the patient was not in a horizontal position (i.e., sitting, head up, etc.). Less frequently (14.5%), PH was used in combination with other techniques to reduce the blood loss (see Table 1). Techniques used to decrease the risks of PH included hydration, maintaining adequate hematocrit, intraoperative monitoring, limiting the time of PH, hypothermia, and others. Various drugs were reportedly used to induce and maintain PH (Figure 11).

## Discussion

Permissive hypotension had been used for decades in neurosurgery in an attempt to reduce intraoperative blood loss, lower blood transfusion rates, and improve operative conditions. With time, potential adverse effects and limitations of PH were better understood. Increased concerns for cerebral ischemic damage, cardiovascular complications, and kidney dysfunction attributable to PH reduced its role in clinical practice. Furthermore, long term outcomes of using PH have never been reported. Nevertheless, the method was not abandoned completely and continued to be used, although with less popularity, by some anesthesiologists.

There is published evidence supporting the use of PH in initial management of multi-trauma patients including those with head injury and in cases with aortic aneurysm rupture (1, 2). The method has also proved its efficacy in maxillofacial surgery (3, 4). Stability of cerebral oxygenation was demonstrated during anesthetic-induced arterial hypotension (5).

Kertai and colleagues conducted a retrospective analysis of the clinical data of 16,263 patients who underwent non-cardiac surgery. They did not find any significant association between intermediate-term mortality and cumulative duration of low MBP below 75 mmHg (6). However, when that parameter was combined with cumulative duration of low bispectral index in a logistic regression model after adding the Cleveland Clinic Risk Index Score and age, low MBP showed a significant correlation with the risk of 30-day mortality (6).

There are many studies that highlight the potential risks and detrimental effects of intraoperative arterial hypotension on end-organ function and treatment outcome. Walsh and colleagues analyzed the data of 33,330 non-cardiac surgeries and found that even short durations of an intraoperative MBP <55 mmHg were associated with acute kidney injury and myocardial injury (7). Sessler et al. concluded that low minimal alveolar concentration of the anesthetic combined with a low MBP, and low bispectral index is an ominous predictor of excessive hospital length of stay and postoperative mortality (8).

Sun and colleagues conducted a retrospective cohort study in 5,127 patients undergoing non-cardiac surgery and described the association between intraoperative hypotension and acute kidney injury (9). The findings suggested an increased risk of postoperative acute kidney injury when the intraoperative MBP was <60 mmHg for over 20 min and <55 mmHg for more than 10 min. However, the study included patients with variable premorbid conditions undergoing different types of procedures who had diverse causes of intraoperative hypotension. With such variability within the group, it would be difficult to extrapolate the data on elective neurosurgical cases undergoing PH under a well-controlled setting.

Monk and colleagues conducted a retrospective cohort study to analyze the association between intraoperative arterial hypotension and hypertension and 30-day postoperative mortality in patients who underwent non-cardiac surgery (10). The intraoperative hypotension, according to the authors, correlated with the risk of increased 30-day mortality. As with the above described study, these results cannot be unequivocally generalized for elective neurosurgical cases. Only 5.6% of the 18,756 patients had neurosurgical procedures. The study included patients with disseminated cancer, patients undergoing peripheral vascular surgery, and emergency cases. Many patients included in this study would not be proper candidates for PH. In addition, the mean age of the study group was 59.5 years, and subjects were predominantly male (92.8%). All these factors make the study conclusions less applicable to patients undergoing elective neurosurgical procedures with application of PH, and we think that this is a limitation of any large-scale database analyses that include multiple surgical pathologies and premorbid conditions.

Thus, significant controversy exists regarding the use of PH in elective neurosurgery. While intraoperative arterial hypotension is, in general, considered deleterious, some anesthesiologists continue to safely use PH in elective neurosurgery.

The results of our survey indicate that, despite heavy criticism, PH is still being occasionally used in modern day neuroanesthesia practice, and the main area of its application is neurovascular surgery. Those respondents who refrain from using PH in their practice, presumably, take into consideration the above discussed potential detrimental effects of the method or use alternative methods to avoid major intraoperative bleeding. Specific requests by the surgical team and local preferences are additional contributing factors to decision-making.

The vast majority of our respondents were from North America, Europe, and Australia. Most of them had significant experience in neurosurgical anesthesia and worked in major hospitals dealing with a large number of neurosurgical patients (Figures 3 and 4). Therefore, it seems reasonable to assume that they used similar standards of patient care, and no bias had been introduced in the study related to substandard health care.

According to the survey results, the main area where PH is still being used is cerebrovascular surgery (Figure 5). Procedures performed on cerebral vessels carry the risk of acute and massive blood loss, intraoperative brain swelling, and flooding the operative field, which may make the surgical control of bleeding problematic. In such circumstances, prophylactic or therapeutic PH may be lifesaving, and its immediate benefits may outweigh the well-known risks.

The detrimental effects of PH correlate with its extent and duration. We think that clinical judgment and experience with the method can help to maximize the benefits of PH and avoid its complications. Coordination with the stage of the surgical procedure, meticulous neurophysiological monitoring, and reasonable limitation of duration and extent of PH are prerequisites to reduce the risk of ischemic damage to the brain and other vital organs. The role of surgical technique cannot be overestimated in minimizing the duration of PH and preventing retraction ischemia during PH.

The majority of survey respondents who use PH prefer a moderate level of hypotension with ~20% decrease of SBP or MBP. On the other hand, multiple reports indicate that short periods of extreme hypotension or blood flow cessation can safely be used in selective cases without causing neurological deterioration or increasing cardiac morbidity (11–14). For example, adenosine can be safely used in cases when temporary clipping of the feeding vessel is not technically possible or when an aneurysmal rupture occurs during the dissection (11). Guinn et al. used 0.24–0.42 mg/kg of adenosine to achieve extreme hypotension defined as SBP <60 mmHg during 30–60 s (12). This time period was usually sufficient to clip the aneurysm. According to the authors, satisfactory operative exposure was achieved in every case. Apparently, the safe time period for using a moderate degree of PH (20–30% less than baseline) will be longer.

At both the Ohio State University Wexner Medical Center and Mayo Clinic, we prefer maintaining the BP at least above the lower normal limits during neurosurgical procedures trying to avoid MBP decrease below 70 mmHg. In some cases, particularly in spinal surgery, we commonly use higher BP target values to ensure adequate perfusion pressure during specific stages of surgery.

Many patients undergoing stereotactic placement of stimulation electrodes for management of Parkinson’s disease are of advanced age and present with preexisting cardiovascular pathologies and chronic arterial hypertension. Another important cause of preoperative arterial hypertension is the white-coat hypertension (15). In this group of patients, at the Ohio State University Wexner Medical Center, we routinely use an infusion of nicardipine or clevidipine to maintain SBP <110 mmHg during the stage of electrode insertion and target localization to minimize pulsatile brain movements and reduce the risk of bleeding. At Mayo Clinic, our goal during similar cases is also to avoid hypertension during deep brain stimulator lead placement. We keep the SBP <140 mmHg by treating with intermittent doses of labetalol if needed. In general, the patients tolerate this slight reduction in SBP for a time period of about 30 min without any neurological or other complications. This correlates with the report of Williams-Russo and colleagues who studied 235 older adults with comorbid medical illnesses undergoing elective primary total hip replacement with epidural anesthesia (16). The authors found no differences in cognitive, cardiac, or renal outcomes in groups with MBP range 45–55 and 55–70 mmHg.

Less frequently, PH had been used by our survey respondents in spinal surgery and transsphenoidal procedures. While significant bleeding is not common in transsphenoidal surgery and usually can be locally controlled, intraoperative bleeding during spinal procedures is mostly of venous origin and induced arterial hypotension during such procedures is not necessarily accompanied by venous or intraosseous hypotension and less blood loss (17). The reduced efficacy along with the risk of spinal ischemia makes PH less applicable for spinal surgery. Similarly, Fearon et al. found no differences in transfusion requirements between hypotensive and normotensive groups of pediatric patients undergoing craniosynostosis correction (18). Again, one possible explanation would be the predominantly venous origin of bleeding in this patient group.

Seventy-three percent of respondents were not aware of any adverse effects directly related to PH in their practice. However, the survey did not include specific questions to clarify postoperative follow-up of the patients by the anesthesia team. In many medical centers, the postanesthesia follow-up is limited to postoperative day 1, which may limit the ability of the anesthesiologist to reveal all possible adverse effects of PH. The most commonly encountered complications of PH were postoperative cognitive dysfunction (POCD) and stroke (Figure 9). However, the questionnaire did not contain any specific questions about POCD, and it was unclear which criteria were used by the respondents to diagnose POCD in their patients and whether any preoperative assessments of the cognitive status were made. Various modifications of EEG, cerebral oximetry, and EP were used by most anesthesiologists to monitor the course of PH (Figure 10). While using modalities of neurophysiological monitoring, one should always keep in mind the limitations of each method including the possibility of false positive and false negative results (19). Nevertheless, multi-modality monitoring during PH is an essential component of anesthesia management and is aimed at reducing the risks of ischemic damage to the neural structures.

Various drugs were used by survey participants to induce and maintain PH (Figure 11). We believe that direct vasodilators including clevidipine, nicardipine, sodium nitroprusside, and others are advantageous in PH management due to their high efficacy, ease of titration, and ability to improve the microcirculatory flow (20–22). Selective calcium channel blockers have the advantage of selective arterial vasodilation with minimal effects on cerebral and spinal perfusion pressures. It is well known that cerebral blood flow is maintained at a lower MBP when hypotension is induced by vasodilators than when it is induced by hemorrhage (23, 24).

Whenever a decision is made to use PH during surgery, certain precautions should be taken to decrease the associated risks. Minimizing the duration and extent of PH, strict coordination of work with the surgeon, multi-modal intraoperative monitoring, and, possibly, pharmacological protection should be used to minimize the risk of adverse events. One of the respondents recommended limiting the MAP decrease to ≤30% baseline for no more than 10–25 min and limiting extreme MAP decrease to ≤ 3–5 min. This time will usually suffice for reaching the surgical goals without a risk of ischemic damage. Optimizing the parameters of PH for various types of neurosurgical procedures can be an area of future research.

### Limitations

Limitations of this survey include the relatively small number of participants and possibility of selection bias due to more frequent participation of anesthesiologists using PH or having special interest in this method. Additionally, given the 13% response rate among SNACC members, it is possible that these findings are not an accurate reflection of practice patterns at all academic medical centers with a high volume neurosurgical practice. Also, the reported complication rate may not accurately reflect the actual incidence of complications related to PH.

## Conclusion

Despite a decline in popularity, PH continues to be used in neuroanesthesia practice by some anesthesiologists with minimal reported early postoperative complication rate. Proper patient selection and adjustment of the extent and duration of arterial hypotension may increase the method’s safety and efficacy, limit the requirements in blood transfusions, and reduce the associated risks of end-organ ischemia. The main area of application for PH in neurosurgical practice remains neurovascular surgery. The fact that PH continues to be used in neuroanesthesia practice justifies a “second look” at the method and further research in this technique to clarify the existing controversies, develop evidence-based recommendations and safer patient-specific strategies for optimal intraoperative BP management in neurosurgical patients.

## Author Contributions

SS, NS, GS, JP, and SB, all contributed equally to the research and writing of this manuscript.

## Conflict of Interest Statement

The authors declare that the research was conducted in the absence of any commercial or financial relationships that could be construed as a potential conflict of interest.

## Abbreviations

AVM, arteriovenous malformation; BP, blood pressure; EEG, electroencephalography; EP, evoked potentials; IH, induced hypotension; MBP, mean blood pressure; SNACC, Society for Neuroscience in Anesthesiology and Critical Care; POCD, postoperative cognitive dysfunction; PH, permissive hypotension; SBP, systolic blood pressure.
